# Effectiveness of Traditional Chinese Medicine as an Adjunct Therapy for Parkinson’s Disease: A Systematic Review and Meta-Analysis

**DOI:** 10.1371/journal.pone.0118498

**Published:** 2015-03-10

**Authors:** Guoxin Zhang, Nian Xiong, Zhentao Zhang, Ling Liu, Jinsha Huang, Jiaolong Yang, Jing Wu, Zhicheng Lin, Tao Wang

**Affiliations:** 1 Department of Neurology, Union Hospital, Tongji Medical College, Huazhong University of Science and Technology, Wuhan, Hubei, China; 2 Department of Neurology, Renmin Hospital of Wuhan University, Wuhan, Hubei, China; 3 Department of Epidemiology and Biostatistics and MOE Key Lab of Environment and Health, School of Public Health, Tongji Medical College, Huazhong University of Science and Technology, Wuhan, Hubei, China; 4 Department of Psychiatry, McLean Hospital, Harvard Medical School, Division of Alcohol and Drug Abuse, and Mailman Neuroscience Research Center, Belmont, Massachusetts, United States of America; 5 Harvard Neuro Discovery Center, Boston, Massachusetts, United States of America; Sun Yat-sen University, CHINA

## Abstract

**Background:**

Idiopathic Parkinson disease (PD) is a common neurodegenerative disease that seriously hinders limb activities and affects patients’ lives. We performed a meta-analysis aiming to systematically review and quantitatively synthesize the efficacy and safety of traditional Chinese medicine (TCM) as an adjunct therapy for clinical PD patients.

**Methods:**

An electronic search was conducted in PubMed, Cochrane Controlled Trials Register, China National Knowledge Infrastructure, Chinese Scientific Journals Database and Wanfang data to identify randomized trials evaluating TCM adjuvant therapy versus conventional treatment. The change from baseline of the Unified Parkinson’s Disease Rating Scale score (UPDRS) was used to estimate the effectiveness of the therapies.

**Results:**

Twenty-seven articles involving 2314 patients from 1999 to 2013 were included. Potentially marked improvements were shown in UPDRS I (SMD 0.68, 95%CI 0.38, 0.98), II (WMD 2.41, 95%CI 1.66, 2.62), III (WMD 2.45, 95%CI 2.03, 2.86), IV (WMD 0.32, 95%CI 0.15, 049) and I-IV total scores (WMD 6.18, 95%CI 5.06, 7.31) in patients with TCM plus dopamine replacement therapy (DRT) compared to DRT alone. Acupuncture add-on therapy was markedly beneficial for improving the UPDRS I–IV total score of PD patients (WMD 10.96, 95%CI 5.85, 16.07). However, TCM monotherapy did not improve the score. The effectiveness seemed to be more obvious in PD patients with longer adjunct durations. TCM adjuvant therapy was generally safe and well tolerated.

**Conclusions:**

Although the data were limited by methodological flaws in many studies, the evidence indicates the potential superiority of TCM as an alternative therapeutic for PD treatment and justifies further high-quality studies.

## Background

Parkinson’s disease (PD) is the second most common progressive neurodegenerative disorder worldwide; it is caused by progressive loss of dopaminergic neurons in the substantia nigra and features physical signs including distal resting tremor, bradykinesia, rigidity, asymmetric onset and non-motor symptoms [1]. However, the exact etiology and pathogenesis of PD remains elusive [2]. In Mainland China, the prevalence of PD over age of 65 years is 1.7% with a rising trend annually [3]. However, no treatment is available to effectively slow down or halt PD progression [4]. Dopamine replacement therapy (DRT) is still the most effective symptomatic strategy, and levodopa is the only drug that has been reported availably to extend life expectancy [5]. After long-term DRT usage, however, the therapeutic effects become increasingly less beneficial, and more than 50% of patients eventually experience highly disabling fluctuations, dyskinesia and agonist-induced sleep attacks [6–9]. In traditional Chinese medicine (TCM), PD symptom was first described as shaking palsy by Huangdi Neijing (ca. 100 A.D.), and TCM has exerted an indispensable effect in medical care of PD patients for thousands of years. Good compliance for long-term use with few side effects may be the foremost merits of TCM. Recently, a meta-analysis demonstrated that Chinese Herb Medicine (CHM) adjuvant therapy improved the clinical symptom severity scores of PD patients with few adverse effects compared to Western Conventional Medication (WCM) controls [10]. TCM is a holistic system of medicine including herbal medicine, acupuncture, moxibustion, Tai Chi, tuina, dietary therapy and qigong. With unique theories of diagnosis and treatment, TCM emphasizes overall treatment concepts based on syndrome differentiation. Up to 2013, a considerable number of clinical trials examining the role of TCM in PD treatment have been performed. The goal of the present study was to assess the efficacy and safety of TCM in PD treatment and to provide additional treatment options for PD patients.

## Materials and Methods

### Data Sources and Search Strategies

We undertook a systematic review to identify randomized clinical trials of TCM that were published before October 2013. A research protocol was drafted and approved by the faculty members. For the literature review, we searched PubMed, Cochrane Controlled Trials Register (CCTR), China National Knowledge Infrastructure (CNKI), Chinese Scientific Journals Database (VIP), and Wanfang data. The search strategies were used as the cross-referenced TCM/Chinese herbal medicine (CHM) and its proprietary names with Parkinson disease, including “zhong yi” (Chinese medicine), “zhong yao” (Chinese herbs), “zhong yi yao” (traditional Chinese medicine), “zhong cheng yao” (Chinese patent medicine), “zhong cao yao” (Chinese herbal medicine),“zhen ci” (acupuncture), “zhen jiu” (acupuncture and moxibustion), “zhong xi yi jie he” (integrated traditional Chinese and western medicine), “an mo” (massage), “tui na” (tuina), “qi gong” (Qigong), “gua sha” (skin scraping therapy), “ba guan” (cupping), “Parkinson’s”, “Parkinson disease”, and “Parkinson’s complication”. No language restriction was applied. Unpublished trials were not included. These journals all publish peer-reviewed manuscripts with open access.

### Inclusion Criteria

(1) ***Types of studies*:** Randomized controlled trials (RCTs) lasting for at least 12 weeks were included.
(2) ***Types of participants*:** Participants were at Hoehn and Yahr (H&Y) stages 1–4 and were of any age or sex with idiopathic PD diagnosed according to the UK Brain Bank criteria [11], Chinese National Diagnosis Standard (CNDS) of 1984 [12] or the CNDS updated version of 2006 [13].
(3) ***Types of interventions*:** TCMs (herbal medicine, patent medicine, acupuncture, moxibustion, tuina, dietary therapy, qigong and other folk therapies) and Chinese medicine integrated with conventional medicine were included if detailed data were available. In all, interventions were any form of TCMs in any dose alone or as adjunct treatment for PD or PD-related complications. Comparisons between different types of TCMs were excluded.
(4) ***Types of outcome measures*:** In addition to the obligatory measurement index of the Unified Parkinson’s Disease Rating Scale (UPDRS) score, following standards of outcome detection might also be included: the British 39-item Parkinson's Disease Questionnaire (PDQ- 39); Parkinson's Disease Quality of Life Questionnaire (PDQL), Webster scale, H&Y staging, adverse events rate, and others. The UPDRS scale is the most commonly used standard consisting of the following four segments: part I (mentation, behavior and mood) addresses mental dysfunction and mood; part II (activities of daily living, ADL) assesses physical function status; part III (motor section) evaluates motor impairment; and part IV (complications) assesses treatment related motor and non-motor complications.

### Exclusion criteria

Quasi-randomized controlled trials, randomized crossover trials, case reports, reviews, and studies concerning secondary Parkinsonism, cell culture or animal experiments were excluded. Case series or clinical trials regarding the efficiency and safety of TCM on PD were also excluded if they 1) included an inappropriate diagnosis standard for PD; 2) had a treatment course lasting less than 12 weeks; 3) did not use the UPDRS score as the outcome measure; 4) were unverified RCT studies; and 5) were duplicate publications.

### Data Extraction and Analysis

The authors (GXZ, NX and ZTZ) independently scanned the titles and abstracts of studies for eligibility and relevance. Potentially relevant papers were retrieved and reviewed for selection according to the inclusion and exclusion criteria. Any discrepancies were resolved by discussion (95% level of agreement) or further evaluated by the third author (TW). For rigorous data collection, a standard data extraction form was used and the items recorded included the authors, year of publication, type of study, participants (sample size, gender proportion, age and dropouts), diagnostic criteria, outcome measures, TCM/placebo intervention, side effects, laboratory examination, effective rate and results.

### Quality Assessment (risk of bias)

The risk of bias in relation to the study quality was independently evaluated by two authors (GXZ and ZTZ). Disagreements were resolved by discussion, and a level of 95% agreement was achieved. According to the Cochrane Collaboration’s risk of bias assessment tool, items that referred to the assessment of the risk of bias for studies might be related to the method of randomization, concealment of treatment allocation, blinding of patients, researchers and assessors (intervention, data collection and analysis), outcome measures (completeness of outcome data, selective report of outcomes, adequacy of follow-up, and parallel between cases and controls) and any other potential sources of bias.

### Data Synthesis and Analysis

To evaluate the TCM efficiency in controlling PD symptoms versus placebo comprehensively, we synthesized the results in a meta-analysis. Fixed-effect model or random-effect model would be used based on heterogeneity in different trials. Weighted mean difference (WMD) with 95% confidence intervals (CI) was calculated for continuous data using RevMan 5.2 software. Heterogeneity was tested using a standard chi-square test and I2 statistic. Two-tailed P values less than 0.05 were considered statistically significant. Funnel plot analysis and egger’s test were used to detect Publication Bias.

## Results

### Study selection

**Fig 1:**
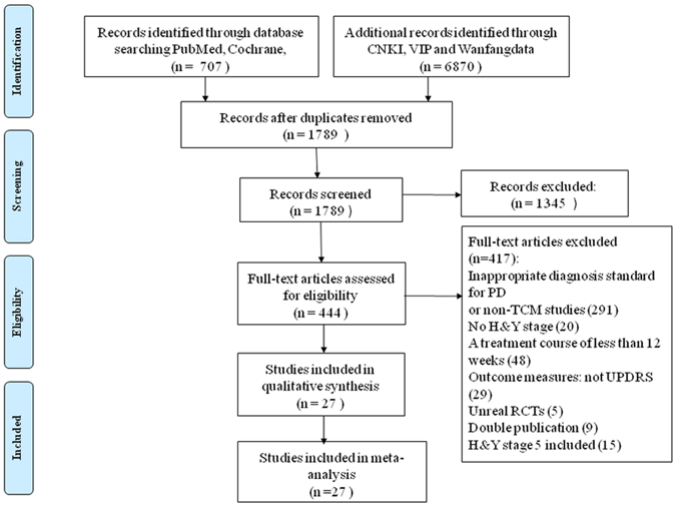
Flow diagram for process of identifying eligible randomized controlled trials. RCT: randomized controlled trials; TCM: Traditional Chinese Medicine; H&Y: Hoehn & Yahr.

By searching CCTR, PubMed, CNKI, VIP, and Wanfang data, we obtained 1789 trials that were potentially relevant to the research project. After reading titles and abstracts of the articles, 1345 trials were excluded due to irrelevant information. For the remaining 444 articles, we performed an overall evaluation, analyzed the full text, and then excluded 417 studies. Finally, 27 papers [14–40] were included in this systematic review. The screening process is summarized in a flow diagram (Fig. 1).

### Characteristics of Included Studies

Of the 27 articles included, 14 employed the 1984 PD diagnostic criteria, 4 used the 2006 PD diagnosis standards (CNDS version updated in 2006), and 9 followed the UK Brain Bank diagnostic criteria. No patient in H&Y stage 5 was included. Except for two studies [26,27] that employed acupuncture, participants in the treatment groups were managed with herbal medicine in the remaining 25 studies, among which two studies [18,20] used TCM as monotherapy and 23 applied TCM plus western medicine as treatment methods.

Among the 27 included studies with a total of 2314 participants (1169 in the treatment group vs. 1145 in the control group), 26 articles demonstrated no significant difference at baseline in gender, age and other basic information. One study, however, reported a distribution difference regarding gender, but with no significant difference observed [33]. Most studies were limited to 12 weeks but in 6 articles the treatment durations lasted for 13 [25] and 24 weeks [17,19, 27,33,40]. Detailed baseline information of the 27 studies is presented in S1 Table. Sixteen articles [14–18,20,22–24,27–31,38,39] described the dosage and frequency of routine therapy. Twenty-five articles reported a clinical curative effect with marked improvement in mental and emotional states, behavioral and daily activities and motor functions of patients as assessed by the UPDRS and other scales, and two trials [18,20] compared TCM alone to Madopar or benzhexol. In toto, 15 studies evaluated the effectiveness of TCM on non-motor symptoms, of which 4 trials reported more effectiveness of TCM on non-motor symptoms than on motor symptoms based on a statistically significant difference (S2 Table). Moreover, 15 articles performed laboratory tests of liver and kidney function and performed electrocardiogram (ECG) experiments showing no obvious abnormalities. Side effects were reported in eight studies [20,22,25,29,31,33,36,37] with 75 vs. 105 person-time (treatment group vs. control group), including diarrhea, nausea, constipation, dry mouth, stomachache, sialorrhea, hypotension, insomnia and depression. Most side effects in the TCM group were relatively mild, occurred less frequently and were less severe than those in the western medicine group and always disappeared after decrement or discontinuation of the drugs. However, three trials reported a high adverse effect frequency in both the treatment and control groups (13 vs. 24 person-time [22], 46 vs. 35 person-time [36], and 17 vs. 29 person-time [37]). The study characteristics are summarized in S3 Table. Of the two articles [26,27] that examined acupuncture and included a total of 98 participants, an improvement was obvious in the acupuncture therapy group. Yang et al. [27] reported that acupuncture plus routine treatment yielded an 89.4% improvement in preventing and controlling PD symptoms in comparison to a 52.6% improvement for routine treatment alone. All characteristics of the included studies are summarized in Table 1.

**Table 1:** Data summary and characteristics of the 27 studies included in meta-analysis.

Author (year)	Intervention		Laboratory examination	Outcome Measures	Clinical effect rate		Adverse effect (n)	
	Control group (drug/ dosage /frequency)	Treatment group (drug/ dosage /frequency)			Treatment group	Control group	Treatment group	Control group
Wang et al. 2013 [20]	Benzhexol 1–2mg, bid	Kangzhen Zhijing Capsule, 15 tables/d	U	UPDRS	88%	52%	3	8
Zhang et al. 2013 [18]	Madopar, 125mg, tid-qid; Chinese herb placebo	Bushen Huoxue Granule, bid; Madopar, 125mg, tid-qid; placebo	Y	UPDRS, the TCM symptom scale	77.80%	81.40%	N	N
Zhao et al. 2013 [33]	Western medicine (N/A); placebo	Western medicine (N/A); Guling Pa’an Granul, 6g, tid	Y	UPDRS III	U	U	3	2
Li et al. 2012 [21]	sinemet/madopar (N/A)	sinemet/madopar (N/A); Jiawei Zhichan Decoction once daily	U	Sleep scale, UPDRS	U	U	U	U
Zhong et al. 2012 [26]	Madopar; previous dosage	Moxibustion, qd,3 times/week; Madopar previous dosage	U	UPDRS, H-Y, Webster, ADL	93.30%	63.30%	U	U
Zhong et al. 2012 [35]	Western medicine (N/A)	Western medicine (N/A); Bushen Huoxue Tongluo Capsule	U	UPDRS, LSIB,PDQ39	U	U	U	U
Kum et al. 2011 [25]	Western medicine (N/A); placebo	Western medicine (N/A); Jia Wei Liu Jun Zi Tang	U	UPDRS-IV, PDQ-39, SF-36, GDS, DSQS	U	U	mild diarrhea: 1	N
Pan et al. 2011 [40]	Western medicine (N/A); placebo	Western medicine (N/A); Zeng-xiao An-shen Zhi-chan2	Y	UPDRS II/III/IV, ADL	improved	No improvement	N	N
Zheng et al. 2011 [16]	Madopar, 125–250mg, tid; placebo	Madopar, 125–250mg, tid; Bushen constant vibration, qd	Y	UPDRS, H-Y	46.67%	16.67%	N	N
Fan et al. 2010 [30]	Madopar, 125mg, tid	Madopar, 125mg, tid; Pabing Recipe II once daily	U	UPDRS	46.67%	16.67%	U	N
Yang et al. 2010 [31]	Madopar, 375–1000mg/d; placebo	BushenHuoxue Granules, bid;Madopar 375–1000mg/d	Y	UPDRS III, muscle tension	U	U	2	6
Yuan et al. 2010 [36]	Western medicine (N/A)	Western medicine (N/A); Shudi Pingchan Decoction	Y	UPDRS II, UPDRS III, H&Y	43.33	16.67	GC: 14;Constipation: 22	GC:10;Constipation:25
Jiang et al. 2009 [22]	Madopar <750mg/d	Madopar <750mg/d; Guiling Pa'an Pill, tid	U	UPDRS	86.66%	63.33%	13 person-time	24 person-time
Zhao et al. 2009 [34]*	Madopa/sinemet (N/A); placebo	Madopar/sinemet (N/A); Guiling Pa’an Capsule	Y	UPDRS total/ II/ III	76.23%	68.30%	U	U
Zhao et al. 2009 [34]&	placebo	Guiling Pa’an Capsule	Y	UPDRS total/ II/ III	67.86%	52%	U	U
Zhao et al. 2009 [34]#	Madopar/sinemet (N/A); placebo	Madopar/sinemet (N/A); Guiling Pa’an Capsule	Y	UPDRS total/ II / III	78.67%	70.89%	U	U
Zhu et al. 2009 [19]	Western medicine (N/A)	Western medicine (N/A); Dingzhen tang, bid	U	UPDRS III, autonomic function	U	U	U	U
Lian et al. 2008 [39]	Madopar, 125mg, tid; placebo	Madopar,125mg tid; Jiaweiguizhijiagegen-tang	Y	UPDRS	U	U	N	N
Liang et al. 2008 [29]	Madopar, 125–250mg, tid-qid; Trastal, 50mg, bid	Madopar, 125–250mg, tid-qid; Five Zhui feng powder, qd	Y	UPDRS	86.67%	70%	N	1
Shen et al. 2008 [17]	Madopar 467 ± 164 mg/ d	Madopar 442 ± 149 mg/d; Tongxinluo Capsule, 9 pills/d; Liuwei Rehmannia Root Pill 24 pills/d	U	UPDRS III	U	U	U	U
Zhang et al. 2008 [23]	Madopar,125mg, tid; placebo	Madopar,125mg,tid; Dingzhen Decoction, qd	U	UPDRS	U	U	U	U
Zhang et al. 2008 [24]	Madopar, 125mg/d	Madopar 125mg/d; Pabing Recipe I/II	Y	UPDRS, H-Y	86.67%	70%	N	N
Lian et al. 2007 [38]	Madopar, 125mg, tid; placebo	Madopar, 125mg, tid; Pabing Recipe I/III, qd	Y	UPDRS-I/II/III/IV/total	Pabing Recipe I 95.5%; III 100%	91.80%	N	N
Luo et al. 2007 [32]	Madopar (N/A)	Madopar (N/A); Pabing Recipe I	U	UPDRS	U	U	U	U
Shen et al. 2006 [37]	Western medicine (N/A)	Western medicine (N/A); Chinese herb medicine	Y	UPDRS II/III, Webster	75	6.3	Vomit: 5; Constipation: 8; Mouth dry: 4	Vomit: 11; Constipation: 13; Mouth dry: 5
Yang et al. 2006 [27]	Madopar, 62.5–500mg, bid-qid	Madopar, 62.5–500mg, bid-qid; Acupuncture, qod	U	UPDRS	89.40%	52.60%	U	U
Zheng et al. 2006 [14]	Madopar, 125–250mg, tid	Madopar, 125–250mg, tid; Pabing Recipe III, once daily	Y	UPDRS, H-Y	43.33%	16.67%	N	N
Wang et al. 2004 [15]	Madopar, 125–250mg, bid; basic therapy (N/A); Placebo	Ziyin Xifeng Huoxue Decoction, qd; basic therapy (N/A)	Y	UPDRS	80%	75.50%	N	N
Zhang et al. 2004 [28]	Madopar(475.0±320.5)mg; Placebo	Madopa(425.5±296.2)mg; naokangning capsule, 3 pills, tid	Y	UPDRS,SF-36, PDTCMS	76.67%	60%	N	N

U: Unclear; Y: yes; N: No; GC: gastrointestinal complaints; UPDRS: unified Parkinson disease rating scale; H-Y: Hoehn and Yahr; ADL: Activities of daily living; LSIB: Life Stisfction lnex B; PDQ39: Parkinson’s Disease Questionnaire-39; SF-36: Short Form-36 Health Survey; PDTCMS: Parkinson’s Disease Traditional Chinese Medicine scale; GDS: Geriatric Depression Scale; DSQS: Deficiency of Spenic Qi Scale. N/A: no detailed information.

*: H&Y stages 4 with Madopar-taking history;

&: H&Y stages 1–4 without Madopar-taking history;

#: H&Y stages 1–3 with Madopar-taking history.

### Risk of Bias of Included Studies

The Cochrane Collaboration’s risk of bias assessment tool was used to assess the risk of bias. Items evaluated included random allocation, concealment schemes, similarity at baseline, placebo, blinding, drop-out, intention-to-treat analysis, acceptable compliance, cross-over trials, and selective reports. Thirteen trials [14,18,24,27,28,30–34,36,38,40] described the stochastic methods in detail including simple random sampling [14,24,30,32], random number table random sampling [18,27,28,38], online random grouping [31,33], random numbers [40], and SAS statistical software [34,36]. Six trials [15,25,31,33,36,40] mentioned allocation concealment, including a central telephone randomization system [31,33], coded containers [25,40], and sealed envelopes [15,36]. Seven studies [18,25,28,31,33,34,40] were designed with blind methods including single blind [18,28] and double blind [25,31,33,34,40] methods. All included studies were parallel, and participants were assessed before and after therapy. However, only nine studies reported relevant information regarding follow-up, of which five trials stated that 74 participants [25,31,33,34,40] from both groups eventually left the study, and four trials [27,30,37,38] indicated no dropouts, while the remaining 20 trials failed to mention withdrawal information. One trial [25] stated their utilization of an intention-to-treat analysis (ITT), and two trials [31,33] reported sample size estimation methods, while the others merely mentioned the number of participants (Table 2).

**Table 2:** The risk information of bias of included trials.

Study	Random allocation	Concealment schemes	Blinding (patient)	Blinding (researcher)	Blinding (assessor)	Intention-to-treat analysis	Drop-out	Similarity at baseline	Placebo	Compliance acceptable	Cross-over trial	Selective report	Jadad Scale
Wang et al. 2013 [20]	n.r	N	N	N	N	n.r	n.r	Y	N	Y	N	N	1
Zhang et al. 2013 [18]	Y	n.r	Y	N	N	n.r	n.r	Y	Y	Y	N	N	2
Zhao et al. 2013 [33]	Y	Y	Y	Y	N	N	Y	N	Y	n.r	N	n.r	7
Li et al. 2012 [21]	n.r	n.r	N	N	N	n.r	n.r	Y	N	Y	N	N	1
Zhong et al. 2012 [26]	n.r	n.r	N	N	N	n.r	n.r	Y	N	Y	N	N	1
Zhong et al. 2012 [35]	n.r	n.r	N	N	N	n.r	n.r	Y	N	n.r	N	n.r	1
Kum et al. 2011 [25]	Y	Y	Y	Y	N	Y	Y	Y	Y	n.r	N	n.r	7
Pan et al. 2011 [40]	Y	Y	Y	Y	N	N	Y	Y	Y	n.r	N	n.r	7
Zheng et al. 2011 [16]	n.r	n.r	N	N	N	n.r	n.r	Y	Y	Y	N	N	1
Fan et al. 2010 [30]	Y	n.r	N	N	N	n.r	N	Y	N	n.r	N	n.r	3
Yang et al. 2010 [31]	Y	Y	Y	Y	N	N	Y	Y	Y	n.r	N	n.r	7
Yuan et al. 2010 [36]	Y	Y	N	N	N	n.r	n.r	Y	N	n.r	N	n.r	4
Jiang et al. 2009 [22]	n.r	n.r	N	N	N	n.r	n.r	Y	N	Y	N	N	1
Zhao et al. 2009 [34]	Y	n.r	Y	Y	N	N	Y	Y	Y	n.r	N	Y	5
Zhu et al. 2009 [19]	n.r	N	N	N	N	n.r	n.r	Y	N	Y	N	N	1
Lian et al. 2008 [39]	n.r	n.r	N	N	N	n.r	n.r	Y	Y	n.r	N	n.r	1
Liang et al. 2008 [29]	n.r	n.r	N	N	N	n.r	n.r	Y	N	n.r	N	n.r	1
Shen et al. 2008 [17]	n.r	n.r	N	N	N	n.r	n.r	Y	N	Y	N	N	1
Zhang et al. 2008 [23]	n.r	n.r	N	N	N	n.r	n.r	Y	Y	Y	N	N	1
Zhang et al. 2008 [24]	Y	n.r	N	N	N	n.r	n.r	Y	N	Y	N	N	2
Lian et al. 2007 [38]	Y	n.r	N	N	N	n.r	N	Y	Y	n.r	N	n.r	3
Luo et al. 2007 [32]	Y	n.r	N	N	N	n.r	n.r	Y	N	n.r	N	n.r	2
Shen et al. 2006 [37]	n.r	n.r	N	N	N	n.r	N	Y	N	n.r	N	n.r	2
Yang et al. 2006 [27]	Y	n.r	N	N	N	n.r	N	Y	N	n.r	N	n.r	2
Zheng et al. 2006 [14]	n.r	n.r	N	N	N	n.r	n.r	Y	Y	Y	N	N	1
Wang et al. 2004 [15]	Y	Y	N	N	N	n.r	n.r	Y	Y	Y	N	N	3
Zhang et al. 2004 [28]	Y	n.r	Y	N	N	n.r	n.r	Y	Y	n.r	N	n.r	2

Y: yes; N: no; n.r: not report;

Jadad scale: Points were determined as follows, I. generation of allocation sequence (computer-generated random numbers, 2 points; not described, 1 point; inappropriate method, 0 point); II. allocation concealment (central randomization, sealed envelopes or similar, 2 points; not described, 1 point; inappropriate or unused, 0 point); III. blindness (identical placebo tablets or similar, 2 point; inadequate or not described, 1 point; inappropriate or no double blinding, 0 point); IV. withdrawals and drop-outs (numbers and reasons are described, 1 point; not described, 0 point). The Jadad scale score ranges from 1 to 7; higher score indicates better RCT quality.

### Synthesis of Results

Among the 27 selected trials, two [26,27] assessed acupuncture plus conventional therapy vs. conventional therapy alone, one [34] assessed TCM vs. a placebo, two [18,20] assessed TCM vs. Western drugs, and 23 evaluated the effect of TCM plus western drugs vs. western therapy alone, of which 9 [14,16,23,30,32,35,38–40], 12 [14,16,23,29,30,32,34–36,38–40], 14 [14,16,19,23,29–32,34–36,38–40], 10 [14,16,23,25,30,32,35,38–40], and 10 [15,21,22,28,32,34,36–38,40] estimated the treatment effect using the UPDRS-I, UPDRS-II, UPDRS-III, UPDRS-IV, and UPDRS-total scores, respectively.

### TCM plus western drugs vs. western therapy alone

**Fig 2:**
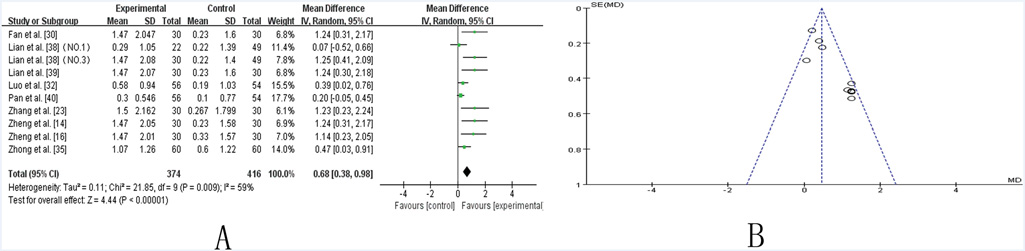
TCM plus western drugs VS. western therapy alone: UPDRS I score. **(A) Forest plot of comparison.** NO.1: Pabing Recipe I; NO.3: Pabing Recipe III. **(B) Funnel plot of comparison.**

***UPDRS-I*.** Nine trials [14,16,23,30,32,35,38–40] compared the effect of TCM plus Western drugs versus Western drugs alone according to changes in the UPDRS-I score. Ten trials showed mild heterogeneity in the consistency of the trial results (chi-square = 21.85, *P* = 0.009; *I2* = 59%). Therefore, a random effects model was used for statistical analysis. A meta-analysis showed a significant beneficial effect of TCM as an adjuvant compared to Western drugs alone in improving the UPDRS-I score (SMD 0.68 [0.38, 0.98]; Z = 4.44, *P* < 0.00001). The effect contributed to controlling the symptoms of mentation, behavior and mood in PD patients (Fig. 2A). The funnel plot was roughly symmetric (Fig. 2B). However, Egger’s test indicated publication bias existed (P = 0.000).

**Fig 3:**
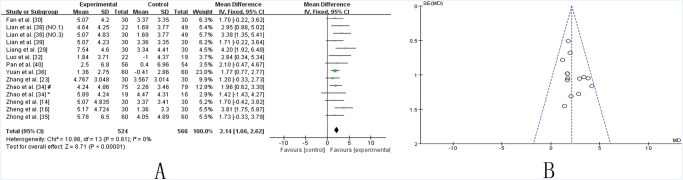
TCM plus western drugs VS. western therapy alone: UPDRS II score. **(A) Forest plot of comparison.** NO.1: Pabing Recipe I; NO.3: Pabing Recipe III; *: H&Y stages 4 with Madopar-taking history; #: H&Y stages 1–3 with Madopar-taking history. **(B) Funnel plot of comparison.**

***UPDRS-II*.** Twelve studies [14,16,23,29,30,32,34–36,38–40] showed homogeneity in the consistency of the trial results (chi-square = 10.98, *P* = 0.61; *I*2 = 0%). A fixed effects model was used. Compared to conventional treatment alone, TCM plus conventional therapy significantly decreased the UPDRS-II scores (WMD 2.41, [1.66, 2.62]; Z = 8.71, *P* < 0.00001), which indicated that TCM might improve the activities of daily life in PD patients (Fig. 3A). The funnel plot was symmetric (Fig. 3B). Egger’s test indicated no publication bias (P = 0.670).

**Fig 4:**
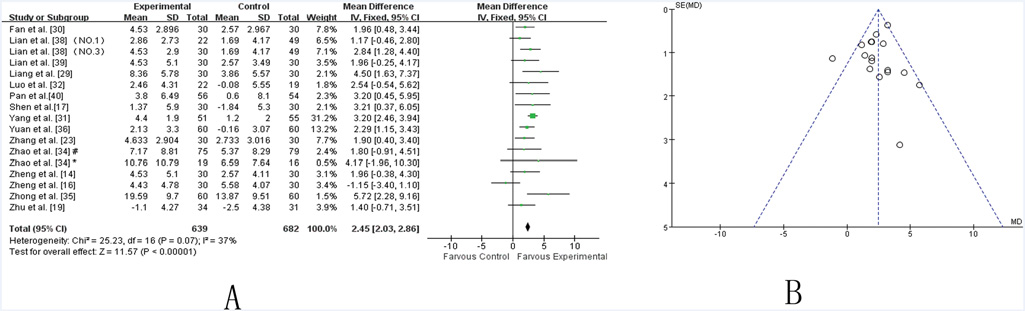
TCM plus western drugs VS. western therapy alone: UPDRS III score. **(A) Forest plot of comparison.** NO.1: Pabing Recipe I; NO.3: Pabing Recipe III; *: H&Y stages 4 with Madopar-taking history; #: H&Y stages 1–3 with Madopar-taking history. **(B) Funnel plot of comparison.**

***UPDRS-III*.** Fourteen independent trials [14,16,19,23,29–32,34–36,38–40] showed mild heterogeneity in the consistency of the results (chi-square = 25.23, *P* = 0.07; *I*2 = 37%). Thus, a fixed effects model was applied. The statistical analysis indicated that adjuvants TCMs were more beneficial than routine methods in alleviating the UPDRS-III score, specifically for motor function (WMD 2.45 [2.03, 2.86]; Z = 11.57, *P* < 0.00001) (Fig. 4A). The funnel plot was symmetric (Fig. 4B). Egger’s test indicated no publication bias (P = 0.630).

**Fig 5:**
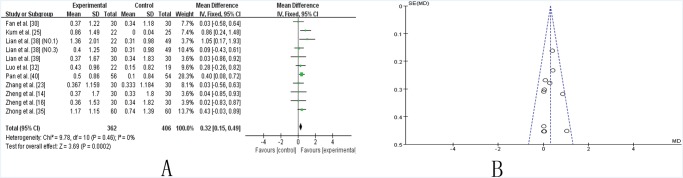
TCM plus western drugs VS. western therapy alone: UPDRS IV score. **(A) Forest plot of comparison.** NO.1: Pabing Recipe I; NO.3: Pabing Recipe III. (**B) Funnel plot of comparison.**

***UPDRS-IV*.** Ten studies [14,16,23,25,30,32,35,38–40] showed homogeneity in the results (chi-square = 9.78, *P* = 0.46; *I*2 = 0%). A fixed-effects model was used. Compared to conventional treatment, CHM adjuvant therapy significantly improved the UPDRS IV scores (WMD: 0.32, [0.15, 049]; Z = 3.69, P = 0.0002), suggesting that CHM adjuvant therapy contribute to improving complications of treatment in patients with PD (Fig. 5A). The funnel plot was symmetric (Fig. 5B). Egger’s test indicated no publication bias (P = 0.788).

**Fig 6:**
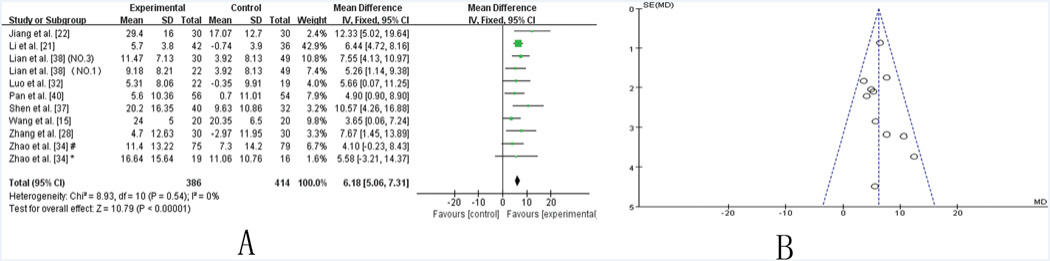
TCM plus western drugs VS. western therapy alone: UPDRS I-IV total score. **(A) Forest plot of comparison.** NO.1: Pabing Recipe I; NO.3: Pabing Recipe III; *: H&Y stages 4 with Madopar-taking history; #: H&Y stages 1–3 with Madopar-taking history. (**B) Funnel plot of comparison.**

***UPDRS I–IV total*.** Ten trials [15,21,22,28,32,34,36–38,40] showed homogeneity in the trial results (chi-square = 8.93, *P* = 0.54; *I*2 = 0%). Therefore, a fixed effects model was applied for statistical analysis. The results showed a significant beneficial effect of TCM as an adjuvant therapy in improving the UPDRS-total score (WMD: 6.18 [5.06, 7.31]; Z = 10.79, *P* <0.00001), suggesting that TCM as an adjuvant therapy markedly improve the symptoms of PD patients (Fig. 6A). The funnel plot was symmetric (Fig. 6B). Egger’s test indicated no publication bias (P = 0.586).

### Acupuncture plus Madopar vs. Madopar alone

Two trials [26,27] evaluated Madopar with or without acupuncture as an adjuvant therapy. No heterogeneity was found (chi-square = 0.94, *P* = 0.33; *I*2 = 0%). Consequently, a fixed model was applied. Acupuncture as an adjuvant therapy combined with Madopar was markedly beneficial in improving the UPDRS I–IV total score in PD patients (WMD: 10.96, [5.85, 16.07]; Z = 4.21, *P* <0.0001) (S1 Fig.).

### TCM vs. placebo

TCM vs. placebo treatment of PD was tested in one study [34]. Compared with the placebo, TCM showed significant improvement in the UPDRS-III score [MD 2.1, (-0.98, 5.18)] (S2A Fig.) and the UPDRS I-IV total score [MD 2.57, (-2.37, 7.51)] (S2B Fig.).

### TCM vs. Western drugs

TCM vs. Western drugs were tested in 2 trials [18,20]. Compared with Madopar, Bushen Huoxue Granules alone did not improve PD symptoms according to the UPDRS I-IV total score [MD-2.3, (-4.1, -0.50)] [18] (S3 Fig.). Similarly, compared with benzhexol, Kangzhen Zhijing capsules alone did not improve the UPDRS-II/III score (data not shown).

### Side effects

**Fig 7:**
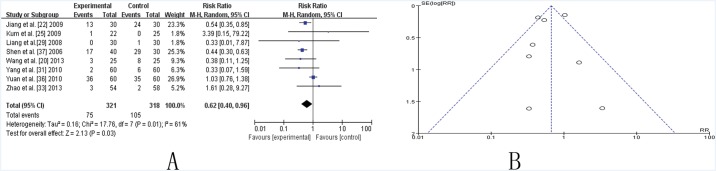
TCM plus western drugs VS. western therapy alone: Side Effect. (A) Forest plot of comparison. (B) Funnel plot of comparison.

Eight studies involving side effect showed mild heterogeneity in the consistency of the results (chi-square = 17.76, *P* = 0.01; *I*2 = 61%). A random-effects model was used for statistical analysis. A statically significant improvement was shown in the RR (0.62, [0.40, 0.96], P (Z) = 0.03), suggesting that CHM adjuvant therapy significantly reduce side effects caused by conventional medicine (Fig. 7A). The funnel plot was roughly symmetric (Fig. 7B). However, Egger’s test indicated that publication bias existed (P = 0.000).

### Studies that lasted 24 weeks vs. those that lasted 12 weeks

Five studies [17,19,27,33,40] that assessed the effectiveness of TCM lasted for 24 weeks and were compared to studies that lasted for 12 weeks. A significant improvement was achieved in two trials [17,19] that lasted for 24 weeks (SMD 4.59, [2.85–6.33], P (Z)<0.00001) (S4A Fig.) compared to those that lasted for 12 weeks (WMD 2.45, [2.03, 2.86], P (Z)<0.00001) (Fig. 4A) with respect to UPDRS III scores. Similarly, a marked improvement in the UPDRS IV score was presented in the study performed by Kum et al. [25] when the results of 24 week studies (MD 2.44, [1.42, 3.46], (Z)<0.00001) (S4B Fig.) were compared to those of 12 week studies (MD 0.32, [0.15–0.49], P (Z) = 0.0002) (Fig. 5A).

### Studies with vs. without randomized and placebo-controlled designs

To further examine any differences between the studies having non-blinding/non-placebo-controlled trials and those with randomized/placebo-controlled designs, we performed a meta-analysis separately. A higher level of effectiveness was found in studies without randomized and placebo-controlled designs by the UPDRS-I, UPDRS-II and UPDRS-total scores, but not by the UPDRS-III and UPDRS-IV scores (S4 Table).

## Discussion

Currently, DRT is still considered the “gold standard” for symptomatic treatment of PD due to the absence of pathological therapies. This systematic review and meta-analysis was designed to summarize the evidence regarding TCM as an adjunct therapy for PD treatment and has shown a stabilizing effect in improving PD motor and non-motor symptoms, as well as reducing the adverse effects of Western medicine. In this meta-analysis, age, gender proportions, types of TCM, course of disease, concurrent disorders and concomitant treatment were all taken into account. Due to the flaws in study designs and the small sample sizes of the studies included in this meta-analysis, the current evidence is insufficient to make a routine recommendation of TCM for PD treatment. However, potentially marked superiorities were shown by Chinese medicine and acupuncture in improving PD patients’ symptoms, as evidenced by the UPDRS scores.

### Evidence of Chinese Medicines

Of all the Chinese medicines adopted in the trials, Pabing Recipe III/ Five Zhui feng powder/ Bushen Huoxue Tongluo Capsule/ Pabing Recipe I/ Guiling Pa'an Pill have been demonstrated to be the most efficacious TCMs in improving UPDRS I/II/III/IV/total scores, respectively (S5 Table). Moreover, we defined a good TCM as it could significantly improve PD symptoms (p<0.05) in at least three aspects of the UPDRS scores. Of all TCMs involved, three TCMs (Pabing Recipe I, Pabing Recipe III, and Zeng-xiao An-shen Zhi-chan 2), which were applied in two studies [38,40], satisfied the standard and could be regarded as good TCMs. The studies lasting for 24 weeks seemed to be more efficacious than those lasting for 12 weeks. However, authenticity was limited due to the small number of studies involved. More clinical trials with larger numbers of participants are necessary to verify our conclusions. In the sensitivity analysis, no change was detected in most studies examined here except for the aspect of side effects. Heterogeneity decreased from 61% to 0% when the study performed by Yuan et al. [36] was excluded. That was likely because PD patients in the Yuan study were older (69.5 ± 7.81 year) and had longer disease durations (7.35 ± 1.82 year) than any other participants in other trials (S1 Table). In fact, TCMs seemed to be well tolerated and had fewer adverse effects in comparison with the conventional medicine controls. Despite of the apparently positive findings, it is still premature to conclude the safety of TCMs for PD treatment because the category and severity of TCMs were not considered in most of the studies that are covered by this meta-analysis. Detailed records and reporting are strongly recommended.

### Evidence of Acupuncture

With respect to the improvements of UPDRS I-IV total scores, acupuncture plus madopar vs. madopar seemed to be the most efficacious with the highest pooled MD (WMD 10.96, 95%CI 5.85–16.07), followed by TCM plus Western drugs vs. Western therapy (WMD 6.18, 95%CI 5.06–7.31), and TCM vs. placebo (MD 2.57, 95%CI-2.37, 7.51). However, only two trials including 117 participants estimated the effect of scalp area and body acupuncture as an adjuvant therapy to madopar. These trials assessed patients’ symptoms immediately before and after therapy but whether any initial improvements persisted for a reasonable period of time was unknown, as no data throughout a follow-up period was available. Collectively, better designs are needed to make consensus in the field as to what form of acupuncture and acupoint is the most appropriate to treat PD patients.

### Limitations

The therapeutic effects should be interpreted with caution due to the methodological weaknesses of the study designs and limitations embodied in the following aspects: ***(*** *1) Randomization* All selected trials demonstrated randomization. However, only 13 trials detailed the randomized grouping methods, while the remaining trials only noted that stochastic grouping methods were used. *(2) Concealment* Six trials applied concealment methods including central telephone randomization systems [31,33], coded containers [25,40], and sealed envelopes [15,36]. Consequently, subjective factors are prone to increase selection bias because researchers tend to assign specific patients into the control or treatment groups. *(3) Placebo and Blinding* Fourteen studies did not use a placebo as a controlled method, and this might discount the placebo effects in the control group. Similarly, the 20 trials that did not use the blinding method tended to induce significant performance and detection bias. *(4) Drop-out* Only one [25] trial adopted the ITT method. Eighteen studies did not mention information regarding drop-outs. The results from these reports might exaggerate the therapy effects. *(5) Heterogeneity* All included trials stated that there were no significant differences at baseline regarding age, gender, and disease course, but seven studies [17,21,23,30,35,38,39] did not report the detailed data. Moreover, the intervention methods and outcome measures were heterogeneous and incomplete.

## Conclusions

Although many experiments are accompanied by flaws in the design methods, the trials included in this meta-analysis do provide potential evidence to indicate that TCM is suitable for long-term use in PD patients with high acceptance and compliance, especially as an alternative medicine for routine therapy. Patients receiving TCM add-on therapy exhibited significant superiorities in PD symptoms as evidenced by improvements in UPDRS scores. Moreover, TCM adjuvant therapy was generally safe, well tolerated, and could significantly reduce the side effects caused by conventional medicine. The effectiveness tended to be more obvious in PD patients with longer adjunct treatment durations. However, to clarify the exact effect of TCM on PD patients, further well-designed studies are needed [41] with the following characteristics: 1) The design should utilize strictly randomized, controlled, double-blind methods; patients should be selected objectively with standard eligibility criteria to reduce the influence of participants, interventions, and outcomes measures, minimizing selective bias, performance bias, and detection bias. 2) Sample sizes should be expanded based on corresponding design formulas. 3) The follow-up should be prolonged to detect a long-term effect. An inadequate follow-up duration in individual trials may significantly influence TCM efficacy. Moreover, laboratory examinations are essential to determine the potential side effects of treatment. 4) Non-motor symptoms of PD should be examined, as the majority of trials are focused on motor symptoms. Moreover, Tai Chi and acupuncture have been shown to markedly improve UPDRS-III scores, selected gait initiation parameters or gait performance, as well as reduce the risk of falling in elderly and frail individuals [42–46].

## Supporting Information

S1 Fig Forest plot of comparison: Acupuncture plus Madopar VS. Madopar alone: UPDRS I-IV total score. (TIF)

S2 Fig Forest plot of comparison: TCM VS. placebo. (A) UPDRS III score. (B) UPDRS I-IV total score.
(TIF)

S3 Fig Forest plot of comparison: TCM VS. Western drugs: UPDRS I-IV total score. (TIF)

S4 Fig Forest plot of comparison: studies lasted 24 weeks VS. those lasted 12 weeks. (A) UPDRS III score. (B) UPDRS IV score.
(TIF)

S1 PRISMA Checklist (DOC)

S1 Table Summary of detailed baseline information of PD patients of included trials. (DOC)

S2 Table *P* value of 15 studies evaluated the effectiveness of TCM on non-motor symptoms. (DOC)

S3 Table Summary of the characteristics of included trials. (DOC)

S4 Table Summary of forest plot of comparison: studies without VS. those with randomized and placebo-controlled designs in respecting to UPDRS I/II/III/IV/total score. (DOC)

S5 Table A list of the most efficacious medicine based on UPDRS scores. (DOC)
