# Supplementary figures and images for: Effectiveness of Traditional Chinese Medicine as an Adjunct Therapy for Parkinson’s Disease: A Systematic Review and Meta-Analysis
Source: PLoS One. 2015 Mar 10;10(3):e0118498. doi: 10.1371/journal.pone.0118498 (PMC4355291; doi:10.1371/journal.pone.0118498)

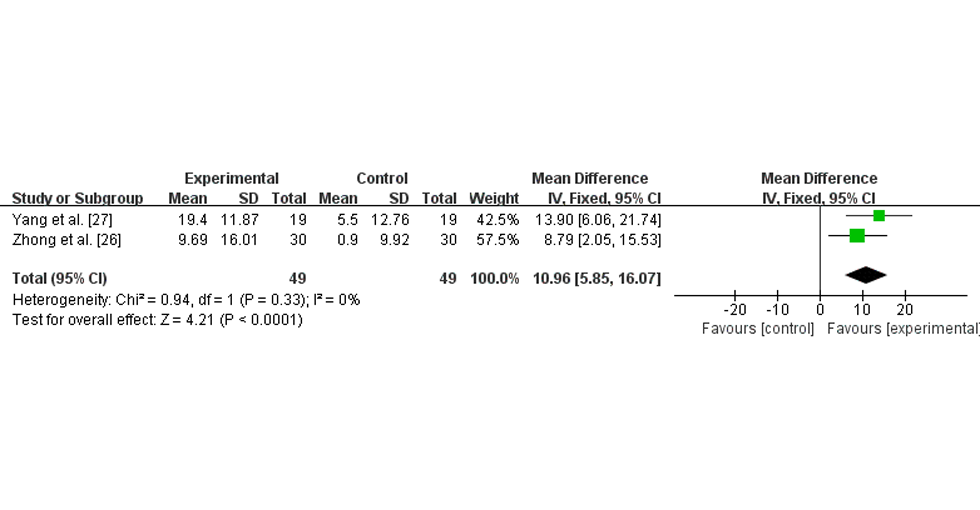

Supplement: S1 Fig — (TIF) [file pone.0118498.s001.tif]

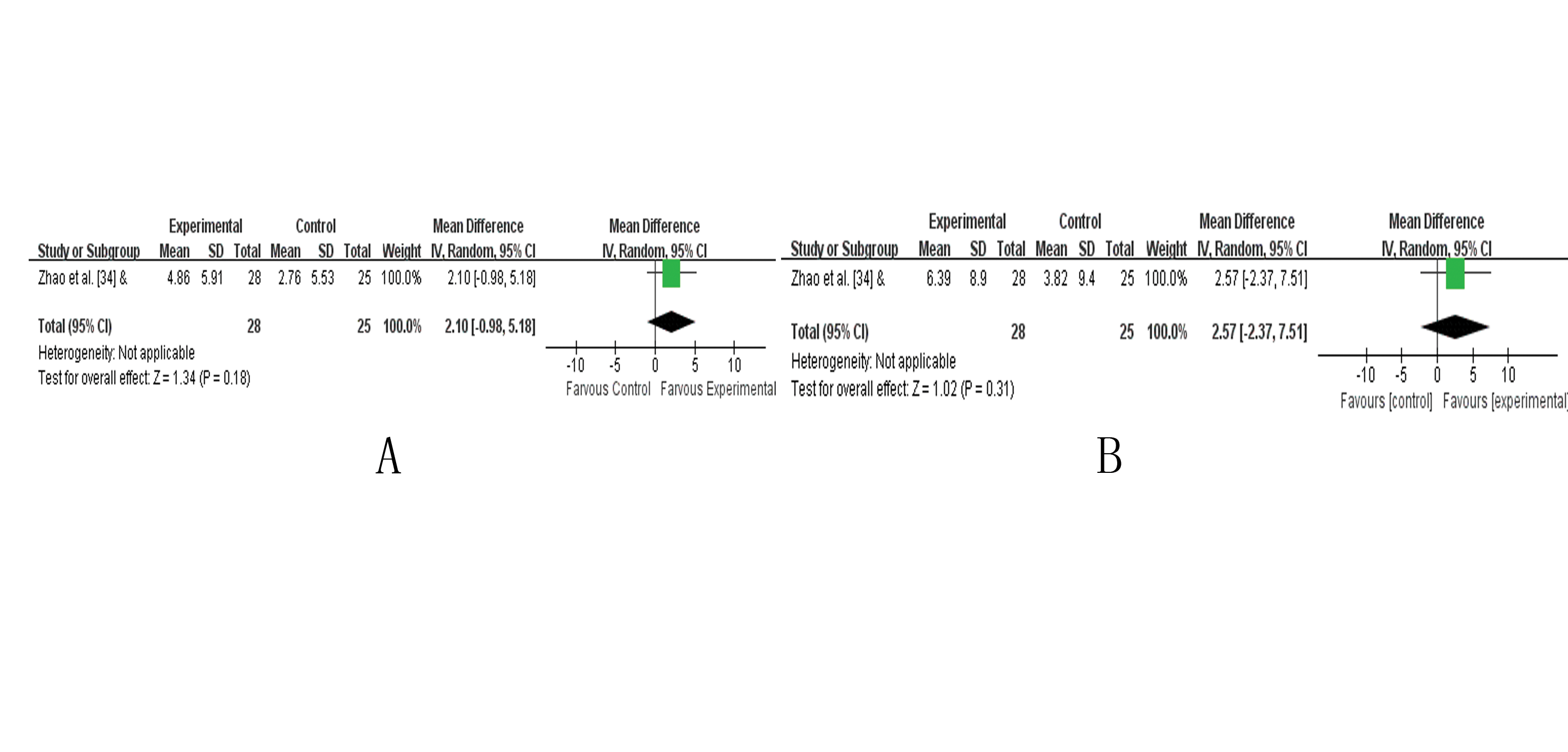

Supplement: S2 Fig — (A) UPDRS III score. (B) UPDRS I-IV total score. (TIF) [file pone.0118498.s002.tif]

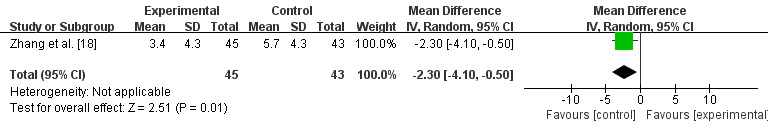

Supplement: S3 Fig — (TIF) [file pone.0118498.s003.tif]

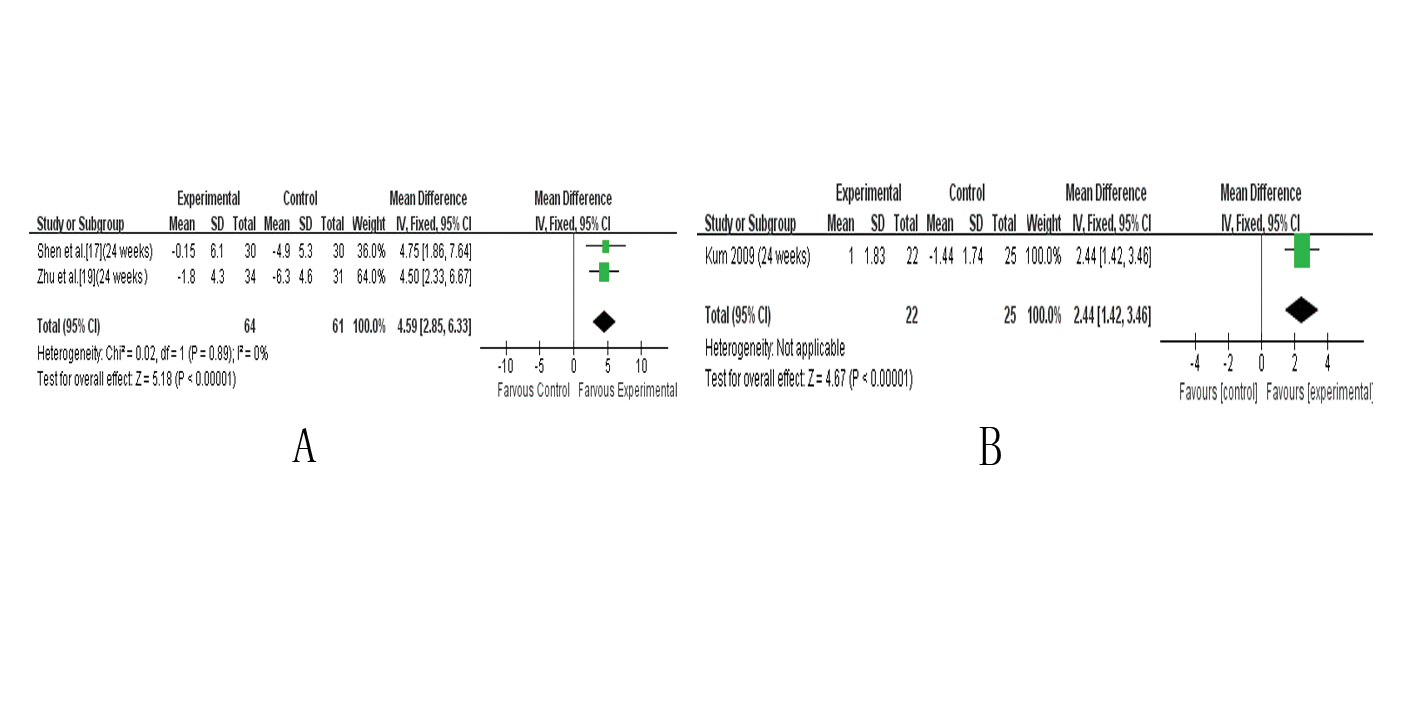

Supplement: S4 Fig — (A) UPDRS III score. (B) UPDRS IV score. (TIF) [file pone.0118498.s004.tif]
