# Supplementary material for: Effectiveness of Traditional Chinese Medicine as an Adjunct Therapy for Parkinson’s Disease: A Systematic Review and Meta-Analysis
Source: PLoS One. 2015 Mar 10;10(3):e0118498. doi: 10.1371/journal.pone.0118498 (PMC4355291; doi:10.1371/journal.pone.0118498)
Supplement: S1 Table — (DOC) [file pone.0118498.s006.doc]

| **Study** | **Case** | **Mean Age (Y)** | | **Gender** | **Course of disease (Year)** | | **Course** | **Diagnostic** |
| --- | --- | --- | --- | --- | --- | --- | --- | --- |
| **Author** | (T/C) | (C) | (T) | (M/F) | (C) | (T) | (Weeks) | Criteria |
| **Wang et al. 2013 [20]** | 25/25 | 55.47±10.64 | 55.47±10.64 | 29/21 | 6.98±2.77 | 6.98±2.77 | 12W | UK |
| **Zhang et al. 2013 [18]** | 45/43 | 63.6±8.1 | 62.1±8.0 | 64/24 | 6.3 ±3.9 | 5.7±4.1 | 12w | UK |
| **Zhao et al. 2013 [33]** | 54/58 | 68.64±8.00 | 68.46士8.80 | 69/43 | 6.39 | 6.79 | 24W | UK |
| **Li et al. 2012 [21]** | 42/36 | 63.50±5.46 | 67.50±6.86 | 22/20 | U | U | 12W | UK |
| **Zhong et al. 2012 [26]** | 30/30 | 61.43±8.33 | 64.53±8.48 | 36/24 | 3.26±2.36 | 4.24±2.93 | 12W | 1984^#^ |
| **Zhong et al. 2012 [35]** | 60/60 | 51-82 | 51-82 | 76/44 | U | U | 12W | UK |
| **Kum et al. 2011 [25]** | 22/25 | 60.88±9.41 | 64.82±8.88 | 31/16 | 5.44±5.26 | 6.37±4.93 | 24W | UK |
| **Pan et al. 2011 [40]** | 59/56 | 62.82±10.31 | 64.7士10.2 | 66/44 | 5.73±4.81 | 5.81±3.24 | 13W | UK |
| **Zheng et al. 2011 [16]** | 30/30 | 62.50±6.92 | 62.53±9.09 | 33/27 | 4.75±3.36 | 4.75±3.5 | 12W | 2006* |
| **Fan et al. 2010 [30]** | 30/30 | U | U | U | U | U | 12W | 2006* |
| **Yang et al. 2010 [31]** | 60/60 | 67.5士9.5 | 66.4±9.1 | 64/42 | 5.3±3.1 | 5.0±3.9 | 12W | UK |
| **Yuan et al. 2010 [36]** | 60/60 | 68.6±7.32 | 69.5士7.81 | 68/52 | 7.43±1.64 | 7.35±1.82 | 12W | 1984^#^ |
| **Jiang et al. 2009 [22]** | 30/30 | 68.33±7.36 | 68.57±7.54 | 33/27 | 4.97±1.50 | 4.70±1.74 | 12w | UK |
| **Zhao et al. 2009 [34]&** | 28/25 | 63.14±11.58 | 65.40 ± 8.16 | 30/23 | 3.69 ± 1.82 | 4.19 ± 3.39 | 12W | 1984^#^ |
| **Zhao et al. 2009 [34]#** | 75/79 | 65.63±8.51 | 64.86 ± 9.85 | 93/61 | 4.27 ± 3.44 | 4.59 ± 3.82 | 12W | 1984^#^ |
| **Zhao et al. 2009 [34]*** | 122/120 | 66.10±7.61 | 67.24 ± 9.54 | 20/15 | 6.24 ± 4.31 | 6.26 ± 2.53 | 12W | 1984^#^ |
| **Zhu et al. 2009 [19]** | 34/31 | 70.0±7.6 | 72.2±6.7 | 44/21 | 3.5 ±2.5 | 3.3 ± 2.4 | 24W | 1984^#^ |
| **Lian et al. 2008 [39]** | 30/30 | 63.9±7.96 | 65.23士9.88 | 42/18 | U | U | 12W | 1984^#^ |
| **Liang et al. 2008 [29]** | 30/30 | 64.6士8.9 | 65.4士9.2 | 35/25 | 11.2±4.1 | 10.6±3.8 | 12W | 1984^#^ |
| **Shen et al. 2008 [17]** | 30/30 | 64.93±8.17 | 64.03±7.49 | 36/24 | U | U | 24W | 2006* |
| **Zhang et al. 2008 [23]** | 30/30 | U | U | U | U | U | 12W | 1984^#^ |
| **Zhang et al. 2008 [24]** | 30/30 | 62.23±6.96 | 64.60±11.78 | 34/26 | 4.25±2.06 | 3.57±2.15 | 12W | 2006 |
| **Lian et al. 2007 [38]** | 52/49 | 63.88±8.58 | 64.5士10.85 | 60/41 | U | U | 12W | 1984^#^ |
| **Luo et al. 2007 [32]** | 22/19 | 66.80±9.15 | 64.54士10.61 | 30/11 | 5.19±5.22 | 5.57±3.56 | 12W | 1984^#^ |
| **Shen et al. 2006 [37]** | 40/30 | 67.91±7.64 | 71.30士6.92 | 46/24 | 4.3±2.31 | 3.91±2.01 | 12W | 1984^#^ |
| **Yang et al. 2006 [27]** | 19/19 | 49-73 | 49-73 | 24/14 | 0.67-6 | 0.67-6 | 14W | 1984^#^ |
| **Zheng et al. 2006 [14]** | 30/30 | 62.30±6.82 | 63.43±10.09 | 34/26 | 4.75±3.42 | 4.75±3.5 | 12W | 1984^#^ |
| **Wang et al. 2004 [15]** | 20/20 | 67.5 | 65.8 | 21/19 | 0.58-5 | 0.58-6 | 12W | 1984^#^ |
| **Zhang et al. 2004 [28]** | 30/30 | 61.58士20.72 | 65.32士18.65 | 34/36 | 5.28±3.44 | 4.98±2.86 | 12W | 1984^#^ |

**S1_Table.** Summary of detailed baseline information of PD patients of included trials.
