# Supplementary material for: Effectiveness of Traditional Chinese Medicine as an Adjunct Therapy for Parkinson’s Disease: A Systematic Review and Meta-Analysis
Source: PLoS One. 2015 Mar 10;10(3):e0118498. doi: 10.1371/journal.pone.0118498 (PMC4355291; doi:10.1371/journal.pone.0118498)
Supplement: S2 Table — (DOC) [file pone.0118498.s007.doc]

| **Author** | **TCM** | **Non-motor** **symptoms** | ***p^a^*** | **Motor symptoms** | ***p^a^*** |
| --- | --- | --- | --- | --- | --- |
| **Li et al. 2012 [21]** | Jiawei Zhichan Decoction | sleep quality; autonomic nervous function | <0.01 | UPDRS-III | >0.05 |
| **Zhu et al. 2009 [19]** | Dingzhen tang | constipation | <0.01 | UPDRS-III | <0.05 |
| **Zhang et al. 2008 [23]** | Ding-Zhen Decoction | UPDRS-I | 0.027 | UPDRS-III | 0.048 |
| **Pan et al. 2011 [40]** | Zeng-xiao An-shen Zhi-chan 2 | nonfluent speech; dysuria; sweating | <0.01 | UPDRS-III | >0.05 |
| **Zhong et al. 2012 [26]** | moxibustion | UPDRS-I | >0.05 | UPDRS-III | <0.05 |
| **Luo et al. 2005 [32]** | Pabing Recipe I | UPDRS-I | 0.081 | UPDRS-III | 0.042 |
| **Lian et al. 2007 [38]** | Pabing Recipe I | UPDRS-I | >0.01 | UPDRS-III | <0.01 |
| **Lian et al. 2008 [39]** | Jiaweiguizhijiageige-Tang | UPDRS-I | >0.05 | UPDRS-III | <0.05 |
| **Fan et al. 2010 [30]** | Pabing Recipe II | UPDRS-I | >0.05 | UPDRS-III | 0.012 |
| **Zhong et al. 2012 [35]** | Bushen Huoxue Tongluo Capsule | UPDRS-I | <0.05 | UPDRS-III | <0.05 |
| **Zheng et al. 2006 [14]** | Pabing Recipe III | UPDRS-I | <0.05 | UPDRS-III | <0.05 |
| **Zheng et al. 2011 [16]** | Bushen Dingzhen tang | UPDRS-I | <0.05 | UPDRS-III | <0.05 |
| **Zhang et al.**  **2004 [28]** | NaoKangNing | constipation; insomnia; dreaminess | <0.05 | rigidity | <0.05 |
| **Kum et al. 2011 [25]** | Jia Wei Liu Jun Zi Tang | UPDRS-IVC | 0.034 | N/A | N/A |
| **Zhao et al. 2013 [33]** | Guling Pa'an Granule | constipation; sweating; insomnia and dreaminess; | <0.0001 | N/A | N/A |

**S2_Table.** *P* value of 15 studies evaluated the effectiveness of TCM on non-motor symptoms.

a, *p* value (compared with control group); N/A, not report.
