# Supplementary material for: Effectiveness of Traditional Chinese Medicine as an Adjunct Therapy for Parkinson’s Disease: A Systematic Review and Meta-Analysis
Source: PLoS One. 2015 Mar 10;10(3):e0118498. doi: 10.1371/journal.pone.0118498 (PMC4355291; doi:10.1371/journal.pone.0118498)
Supplement: S3 Table — (DOC) [file pone.0118498.s008.doc]

| Name of condition | No. of publications (a) | Frequency (a/27) | No. of patients (b) | Frequency (b/2314) |
| --- | --- | --- | --- | --- |
| 1984 PD diagnostic criteria | 14 | 51.85% | 1284 | 55.49% |
| 2006 PD diagnosis standards | 4 | 14.81% | 240 | 10.37% |
| UK Brain Bank diagnostic criteria | 9 | 33.33% | 790 | 34.14% |
| detailed description of randomization methods | 14 | 51.85% | 1451 | 62.70% |
| blind method principle | 7 | 25.93% | 991 | 42.83% |
| >12 weeks | 6 | 22.22% | 437 | 18.89% |
| placebo treatment | 13 | 48.15% | 1372 | 59.29% |
| laboratory examination | 15 | 55.56% | 1575 | 68.06% |
| side effects | 8 | 29.63% | 180 (person-times) | 7.78% |
|  | | | | |

**Table S2. Summary of the characteristics of included trials.**
