# Supplementary material for: Effectiveness of Traditional Chinese Medicine as an Adjunct Therapy for Parkinson’s Disease: A Systematic Review and Meta-Analysis
Source: PLoS One. 2015 Mar 10;10(3):e0118498. doi: 10.1371/journal.pone.0118498 (PMC4355291; doi:10.1371/journal.pone.0118498)
Supplement: S4 Table — (DOC) [file pone.0118498.s009.doc]

**Studies with randomized and placebo-controlled designs**

UPDRS-I
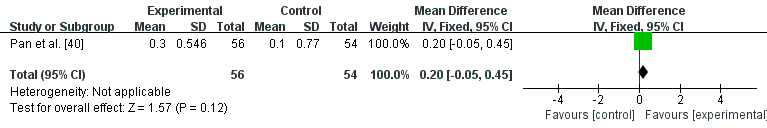


UPDRS-II


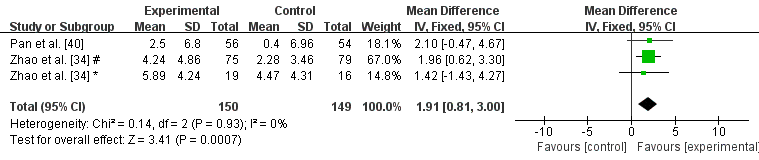


UPDRS-III


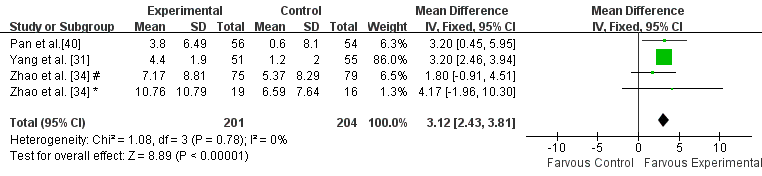


UPDRS-IV


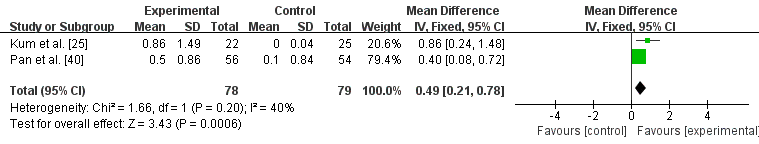


UPDRS I-IV total


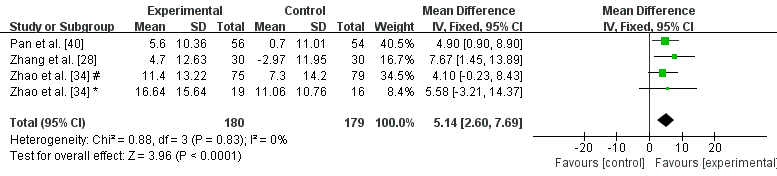


**Studies without randomized and placebo-controlled designs**

UPDRS-I


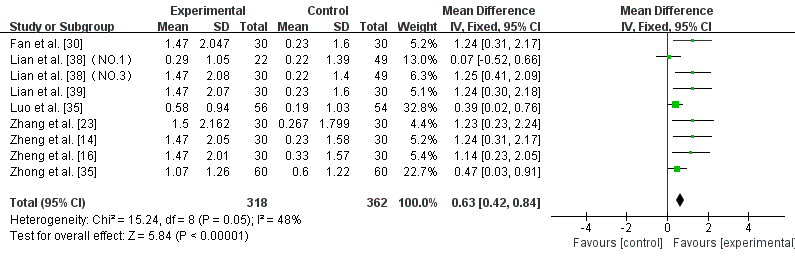


UPDRS-II


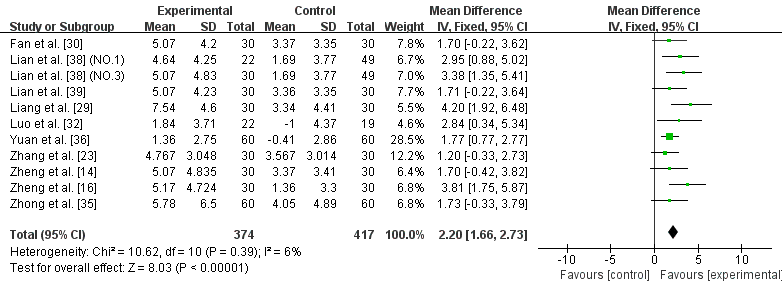


UPDRS-III


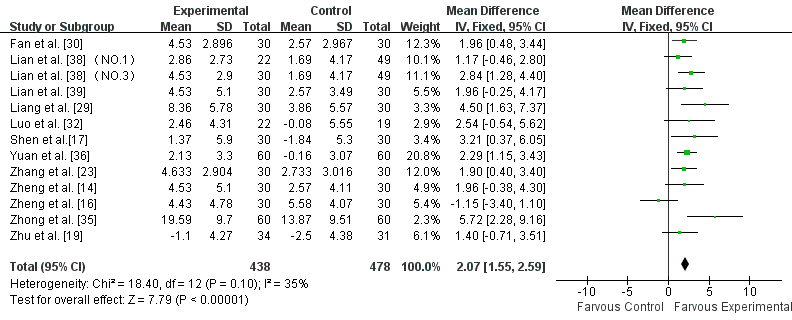


UPDRS-IV


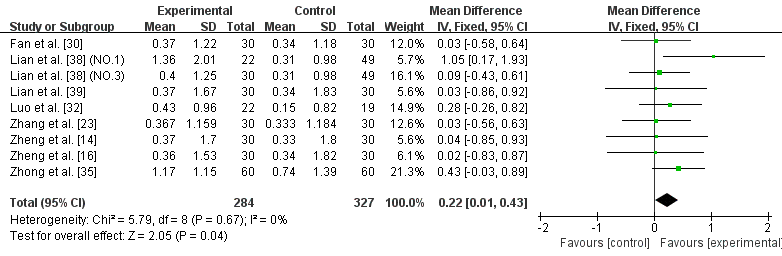


UPDRS I-IV total


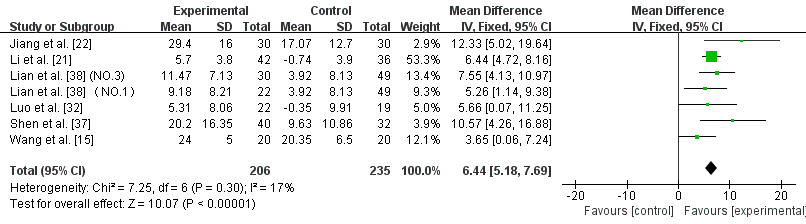


| **Author** | **non-blinding trials** | **blinding trials** |
| --- | --- | --- |
| **UPDRS-I** | WMD 0.63, 95%CI 0.42-0.84, P(Z)<0.00001 | SMD 0.2, 95%CI -0.05-0.45, P(Z)=0.12 |
| **UPDRS-II** | WMD 2.2, 95%CI 1.66-2.73, P(Z)<0.00001 | WMD 1.91, 95%CI 0.81-3.00, P(Z)<0.0007 |
| **UPDRS-III** | WMD 2.07, 95%CI 1.55-2.59, P(Z)<0.00001 | WMD 3.12, 95%CI 2.43-3.81, P(Z)<0.00001 |
| **UPDRS-IV** | WMD 0.22, 95%CI 0.01-0.43, P(Z)=0.04 | WMD 0.49, 95%CI 0.21-0.78, P(Z)<0.0006 |
| **UPDRS-total** | WMD 6.44, 95%CI 5.18-7.69, P(Z)<0.00001 | WMD 5.14, 95%CI 2.6-7.69, P(Z)<0.0001 |

**S4_Table.** Summary of forest plot of comparison: studies without VS. those with randomized and placebo-controlled designs in respecting to UPDRS I/II/III/IV/total score.
