# Supplementary material for: Effectiveness of Traditional Chinese Medicine as an Adjunct Therapy for Parkinson’s Disease: A Systematic Review and Meta-Analysis
Source: PLoS One. 2015 Mar 10;10(3):e0118498. doi: 10.1371/journal.pone.0118498 (PMC4355291; doi:10.1371/journal.pone.0118498)
Supplement: S5 Table — (DOC) [file pone.0118498.s010.doc]

**S5_Table:** A list of the most efficacious medicine based on UPDRS scores

|  | **Author** | **TCM** | | **OR (95%CI)** | | ***P*** | |
| --- | --- | --- | --- | --- | --- | --- | --- |
| **UPDRS-I** | **Lian et al. 2007 [38]** | Pabing Recipe III | 1.25 (0.41, 2.09) | | 0.004 | |  |
| **UPDRS-II** | **Liang et al. 2008 [29]** | Five Zhui feng powder | 4.20 (1.92, 6.48) | | 0.0003 | |  |
| **UPDRS-III** | **Zhong et al. 2012 [35]** | Bushen Huoxue Tongluo Capsule | 5.72 (2.28, 9.16) | | 0.001 | |  |
| **UPDRS-IV** | **Lian et al. 2007 [38]** | Pabing Recipe I | 1.05 (0.17, 1.93) | | 0.02 | |  |
| **UPDRS-total** | **Jiang et al. 2009 [22]** | Guiling Pa'an Pill | 12.33 (5.02, 19.64) | | 0.0009 | |  |
| **Side effects** | **Yang et al. 2010 [31]** | BushenHuoxue Granules | 0.33 (0.07, 1.59) | | 0.17 | |  |
